# A combination of p300 and Braf expression in the diagnosis and prognosis of melanoma

**DOI:** 10.1186/1471-2407-14-398

**Published:** 2014-06-03

**Authors:** Madhuri Bhandaru, Gholamreza Safaee Ardekani, Guohong Zhang, Magdalena Martinka, Kevin J McElwee, Gang Li, Anand Rotte

**Affiliations:** 1 Skin Cancer Biology Laboratory, Department of Dermatology and Skin Science, University of British Columbia, Research Pavilion, 828 West, 10th Avenue, Vancouver, BC V5Z 1 L8, Canada; 2 Department of Pathology & Laboratory Medicine, University of British Columbia, Vancouver, British Columbia, Canada

**Keywords:** p300, Braf, Melanoma, Prognosis, AJCC, Patient survival

## Abstract

**Background:**

To date only a handful of drugs are available for the treatment of melanoma. Among them vemurafenib, a Braf^V600E^ specific inhibitor, showed promising results in terms of response rate and increase in median survival time. However, its effectiveness is limited by development of resistance and the search for additional drugs for melanoma treatment is ongoing. The present study was performed to analyze the correlation between Braf expression and the expression of p300, a known down stream target of the mitogen activated protein kinase (MAPK) pathway, which was recently shown by us to be a prognostic marker for melanoma progression and patient survival.

**Methods:**

The expression of Braf and p300 expression were correlated and analyzed by Chi-square test. A total of 327 melanoma patient cases (193 primary melanoma and 134 metastatic melanoma) were used for the study. Classification & regression tree (CRT), Kaplan-Meier, and multivariate Cox regression analysis were used to elucidate the significance of the combination of Braf and p300 expression in the diagnosis and prognosis of melanoma.

**Results:**

Our results demonstrate that Braf expression is inversely correlated with nuclear p300 and positively correlated with cytoplasmic p300 expression. Braf and cytoplasmic p300 were found to be associated with melanoma progression, tumor size and ulceration status. CRT analysis revealed that a combination of Braf and p300 expression (nuclear and cytoplasmic), could be used to distinguish between nevi and melanoma, and primary from metastatic melanoma lesions. The combination of Braf and nuclear p300 was significantly associated with patient survival and nuclear p300 was found to be an independent predictor of patient survival.

**Conclusion:**

Our results indicate a cross-talk between Braf and p300 in melanoma and demonstrate the importance Braf and p300 expression in the diagnosis and prognosis of melanoma.

## Background

Melanoma, a type of cancer caused due to uncontrolled proliferation of melanocytes in epidermis of skin, is one of the most frequent cancers in fair skinned populations [1,2]. According to recently published statistics based on data from United States of America, it is the fifth most common cancer in men and seventh most common cancer in women [3]. Melanoma is known for its rapid progression, metastasis, and poor prognosis, and is responsible for over 80% of deaths from skin cancer [1]. Early diagnosis allows for surgical excision of the tumors and the patients can be managed with a relapse free interval of up to 10 years [4,5]. But, approximately 1 in 35 patients develop metastatic tumors, and metastatic melanoma has a very poor prognosis with an overall survival between 8 to 18 months. Only 15% of patients with metastatic melanoma survive for 5 years [3,6].

There has been limited progress in the treatment of melanoma; metastatic melanoma is notorious for its resistance to conventional radiotherapy and chemotherapy. Until recently, dacarbazine, a DNA alkylating agent, was the only FDA approved drug available for the treatment of melanoma [6]. In 2011, vemurafenib, a specific inhibitor of BrafV600E (BRAF harbouring a point mutation resulting from a substitution of valine at amino-acid 600 with glutamine), and ipilimumab, a monoclonal antibody against cytotoxic T-lymphocyte associated antigen 4 (CTLA-4), have been approved for the treatment of melanoma [6]. However, the success of their use is limited by effectiveness only in a restricted population, potential development of lethal resistance with vemurafenib treatment, and only a small increase in median survival time in the case of ipilimumab [6]. Our lab previously reported a significant association between increased Braf expression and melanoma progression, and an inverse relationship between Braf expression and patient prognosis [7,8]. Considering the significance of Braf inhibitors in melanoma treatment, several studies have attempted to decipher the mechanisms for resistance and suggested both mitogen activated protein kinase (MAP kinase) dependent and independent pathways as reasons for vemurafenib resistance [6]. A number of strategies to overcome the resistance, including a combination therapy of Braf and MEK1/2 inhibitors, have been proposed and are in various stages of clinical studies [6]. However, there are no results on the efficiency of the combination therapies in clinical settings and the search for alternative and additional drugs for the treatment of melanoma is ongoing.

We analyzed the expression of p300, a well studied histone acetyl transferase (HAT) [9], in melanoma patient samples and found that loss of p300 expression in the nucleus was correlated with disease progression and worse survival in melanoma patients [10]. Furthermore, we also found that nuclear p300 expression was an independent prognostic factor, suggesting the importance of targeting the functions of histone acetyltransferases (HAT) in melanoma therapy [10]. Stability and activity of p300 protein have been shown to be regulated by phosphorylation, and phosphorylation of p300 by mitogen activated protein kinase (MAPK) and extracellular signal-regulated kinase (ERK1/2) has been reported to promote the degradation of p300 protein [11,12]. Since our previous studies in melanoma patients showed an increase in Braf expression, which is known to be upstream of MAPK in the signaling cascade, we hypothesized a potential for correlation between p300 and Braf [8]. To test our hypothesis, and to explore the possible opportunity of targeting histone acetylation and Braf in melanoma treatment, we studied the association between p300 and Braf expression in patient samples.

## Methods

### Patient specimens and tissue microarray construction

The collection of patient specimens and the construction of the tissue microarray (TMA) have been previously described [13]. Briefly, we used patient data collected from 1990 to 2009. Of 748 patients specimens collected, 369 biopsies including 327 melanoma cases (193 primary melanoma and 134 metastatic melanoma) and 42 cases of nevi (21 normal nevi and 21 dysplastic nevi) could be evaluated for comparing p300 and Braf staining in this study, due to loss of biopsy cores or insufficient tumor cells present in the cores. The demographic characteristics of melanoma patients are detailed in Table 1. All specimens were obtained from the archives of the Department of Pathology, Vancouver General Hospital. The use of human skin tissues and the waiver of patient consent in this study were approved by the Clinical Research Ethics Board of the University of British Columbia [14]. The study was conducted according to the principles expressed in the Declaration of Helsinki.

**Table 1:** Demographics and clinical characteristics of 327 melanoma patients

**Variables**	**Total**	**Percentage**
All melanoma		
Age		
≤ 62	166	50.8%
> 62	161	49.2%
Gender		
Male	196	59.9%
Female	131	40.1%
AJCC		
I	80	24.5%
II	113	34.6%
III	55	16.8%
IV	79	24.2%
Primary melanoma (n = 193)		
Age		
≤ 62	89	46.1%
> 62	104	53.9%
Gender		
Male	109	56.5%
Female	84	43.5%
Thickness		
≤ 2.0 mm	91	47.2%
> 2.0 mm	102	52.8%
Ulceration		
Absent	144	74.6%
Present	49	25.4%
Metastatic melanoma (n = 134)		
Age		
≤ 62	77	57.5%
> 62	57	42.5%
Gender		
Male	87	64.9%
Female	47	35.1%

From the original tissue biopsies, the most representative tumor area was carefully selected and marked on hematoxylin and eosin stained slides. Tissue cores of 0.6-mm thickness were taken in duplicate from each biopsy and the TMAs were assembled using a tissue-array instrument (Beecher Instruments, Silver Spring, MD). Using a Leica microtome, multiple 4 μM sections were cut and transferred to adhesive-coated slides using regular histological procedures. One section from each TMA was routinely stained with hematoxylin and eosin while the remaining sections were stored at room temperature for immunohistochemical staining.

### Immunohistochemistry

Tissue microarray (TMA) slides were dewaxed at 55°C for 20 min followed by three 5 min washes with xylene. The tissues were then rehydrated by washing the slides for 5 min each with 100%, 95%, 80% ethanol and finally with distilled water. The slides were then heated to 95°C for 30 min in 10 mmol/L sodium citrate (pH 6.0) for antigen retrieval and then treated with 3% hydrogen peroxide for 1 hour to block the endogenous peroxidase activity. After blocking the slides with the universal blocking serum (Dako Diagnostics, Carpinteria, CA, USA), the sections were incubated overnight with monoclonal mouse anti-p300 antibody (1:50 dilution; Millipore, USA) or with mouse polyclonal anti-Braf antibody (1:100 dilution; Sigma, USA) at 4°C. The sections were then incubated for 30 min with a biotin-labeled secondary antibody and then with streptavidin-peroxidase (Dako Diagnostics). The samples were developed by treatment with 3,3′-diamino-benzidine substrate (Vector Laboratories, Burlington, Ontario, Canada) and with hematoxylin to counter-stain the nuclei. Negative controls were done by omitting the p300/Braf antibody during the primary antibody incubation.

### Evaluation of immunostaining

The evaluation of p300 and Braf staining was done blindly by microscopic examination of the tissue sections by one dermatopathologist and two other observers simultaneously, using a multiple viewing microscope and a consensus was reached for the score of each core. p300/Braf staining intensity was scored as 0+, 1+, 2+, 3+ whereas the percentage of p300/Braf positive cells was scored as 1 (1-25%), 2 (26-50%), 3 (51-75%) and 4 (76-100%). In cases of discrepancy between duplicated cores, the higher score from the two tissue cores was taken as the final score. The product of intensity and percentage was taken as the immunoreactive score (IRS) [15]. Based on IRS, p300 & Braf staining in the tissue sections was categorized as negative (IRS 0), weak (IRS 1–4), moderate (IRS 6–8), or strong (IRS 9–12). Since p300 was found to be expressed in both nucleus and cytoplasm [10], the nuclear and cytoplasmic staining was evaluated in parallel at the same time. The choice of the optimum cut-off values for the IRS were derived based on the IRS pattern in nevi and melanoma cases and are described previously [7,10].

### Statistical analysis

Correlation between p300 and Braf, and clinicopathologic parameters was evaluated by Chi-square test among the patient subgroups. Survival time was calculated from the date of melanoma diagnosis to the date of death or last follow-up. The effect of p300 and Braf on the overall and disease-specific survival was evaluated by Kaplan-Meier analysis and log-rank test. Additionally, multivariate Cox proportional hazards regression models were preformed to estimate the hazard ratios (HRs) and their 95% confidential intervals (CIs). Classification tree was constructed by the classification and regression tree (CRT) model as described previously to examine possibility of using a Braf and p300 combination to identify different stages of melanoma [16]. The decision trees depicting the classification rules were generated through recursive partitioning. When growing each tree, equal prior probabilities to the normal and cancer cohorts, and equal misclassification costs were assigned. To assess the amount of over-fitting, 10-fold cross-validation experiments was performed using the SE rule as described previously [16]. *P*-value <0.05 was considered as statistically significant. All the statistical analyses were performed using SPSS version 16.0 (SPSS Inc, Chicago, IL) software.

## Results

### Braf expression correlates inversely with nuclear p300 and directly with cytoplasmic p300 expression

**Figure 1:**
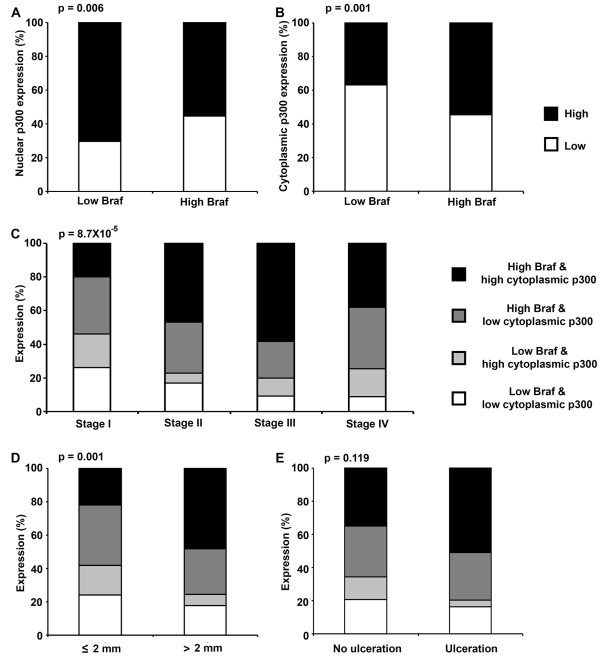
**Braf expression correlates with p300 expression in melanoma patients. (A)** Negative correlation between Braf and nuclear p300 expression in melanoma patient biopsies. Melanomas which have high Braf expression have a significantly higher percentage of low nuclear p300 staining (p = 0.006, χ^2^ test). **(B)** Positive correlation between Braf and cytoplasmic p300 expression in melanoma patient biopsies. Melanomas which have high Braf expression also have a significantly higher percentage of high cytoplasmic p300 staining (p = 0.001, χ^2^ test). High Braf and high cytoplasmic p300 expression is significantly associated with AJCC progression **(C)** and tumor size **(D)**, but not with ulceration status **(E)**. p-values, 8.7×10^-5^, 0.001 & 0.119 respectively (χ^2^ test).

Previous studies showed that phosphorylation by MAP kinase resulted in accelerated degradation of p300 in cardiac cells [11]. Since Braf is known to be an up stream kinase in the MAP kinase pathway, we asked if its expression could be inversely associated with p300 expression in the tumor samples from melanoma patients. Based on the previously reported cut-off values for immunoreactive scores (IRS), we divided the staining into low (IRS 0 to 4) and high (IRS 6 to 12), and matched the expression of Braf and p300 in the melanoma patients [7,10]. Chi-square analysis of the matched data revealed that Braf expression inversely correlated with nuclear p300 and directly correlated with cytoplasmic p300 expression suggesting Braf negatively regulates the nuclear accumulation of p300 (Figure 1A & B).

### Braf and cytoplasmic p300 expression are associated with disease progression

We next asked if the association between Braf and p300 expression was particularly correlated with disease progression or tumor size or ulceration status. We first divided the data based on American Joint Committee for Cancer (AJCC) staging and performed Chi-square test analysis. As shown in Table 2, the percentage of patients with high Braf expression or high cytoplasmic expression was significantly increased as melanoma progressed from AJCC stage I to stage III and then slightly decreased from stage III to stage IV. Accordingly, the percentage of patients with high Braf and high cytoplasmic p300 expression was significantly increased from AJCC stage I through stage III and slightly decreased from stage III to stage IV (Figure 1C). Interestingly, the difference in percentage of patients with high Braf and high cytoplasmic p300 expression was highest between stage I and II, which differ mainly based on the tumor size (Figure 1C) [17]. On the other hand, increase in the percentage of cases with high Braf and low nuclear p300 expression was more apparent between stages II and III, which differ based on the presence of tumor cells in the lymph nodes, an indicator of migration and metastasis (Table 2) [17].

**Table 2:** Correlation between Braf/p300 staining and AJCC stage in 327 melanoma patients

	**Stage I**	**Stage II**	**Stage III**	**Stage IV**	*p*-value*
**Braf**					
Low	37 (46.3%)	26 (23.0%)	11 (20.0%)	20 (25.3%)	9.8 × 10^-4^
High	43 (53.8%)	87 (77.0%)	44 (80.0%)	59 (74.7%)	
**Nuclear p300**					
Low	29 (36.3%)	47 (41.6%)	30 (54.5%)	33 (41.8%)	0.204
High	51 (63.7%)	66 (58.4%)	25 (45.5%)	46 (59.2%)	
**Cytoplasmic p300**					
Low	48 (60.0%)	53 (46.9%)	17 (30.9%)	36 (45.6%)	0.011
High	32 (40.0%)	60 (53.1%)	38 (69.1%)	43 (54.4%)	
**Braf and nuclear p300**					
Low braf low p300	11 (13.8%)	13 (11.5%)	6 (10.9%)	6 (7.6%)	0.010
Low braf high p300	26 (32.5%)	14 (12.4%)	6 (10.9%)	16 (20.3%)	
High braf low p300	18 (22.5%)	34 (30.1%)	24 (43.6%)	27 (34.2%)	
High braf high p300	25 (31.3%)	52 (46.0%)	19 (34.6%)	30 (38.0%)	
**Braf and cytoplasmic p300**					
Low braf low p300	21 (26.3%)	19 (16.8%)	5 (9.1%)	7 (8.8%)	8.7 × 10^-5^
Low braf high p300	16 (20.0%)	7 (6.2%)	6 (10.9%)	13 (16.5%)	
High braf low p300	27 (33.7%)	34 (30.1%)	12 (21.8%)	29 (36.7%)	
High braf high p300	16 (20.0%)	53 (46.9%)	32 (58.2%)	30 (38.0%)	

*- χ^2^ test.

Next we separated the cases based on tumor size (≤2 mm versus >2 mm) and then based on ulceration status (no ulceration versus ulceration). Braf expression was found to be significantly associated with tumor size and ulceration status, whereas cytoplasmic p300 expression was associated with tumor size but not with ulceration status (Table 3). Nuclear p300 expression was not associated with tumor size or ulceration status (Table 3). As seen with melanoma progression, the incidence of larger tumors was significantly higher (Figure 1C), and presence of ulcerated tumors tended to be higher (Figure 1D), in patients with high Braf and high cytoplasmic p300 expression. Though patients with low nuclear p300 tended to be associated with advanced stages of melanoma, larger tumor size and presence of ulcerated tumors, the difference did not reach statistical significance (Table 3).

**Table 3:** Correlation between Braf/p300 staining and tumor size, and ulceration status in 327 melanoma patients

	**Tumor size**		
	**≤ 2 mm**	**> 2 mm**	*p*-value*
**Braf**			
Low	38 (41.8%)	25 (24.5%)	0.011
High	53 (58.2%)	77 (75.5%)	
**Nuclear p300**			
Low	33 (36.3%)	43 (42.2%)	0.403
High	58 (63.7%)	59 (57.8%)	
**Cytoplasmic p300**			
Low	55 (60.4%)	46 (45.1%)	0.033
High	36 (39.6%)	56 (54.9%)	
**Braf and nuclear p300**			
Low Braf and low p300	11 (12.1%)	13 (12.7%)	0.035
Low Braf and high p300	27 (29.7%)	13 (12.7%)	
High Braf and low p300	22 (24.2%)	30 (29.4%)	
High Braf and high p300	31 (34.1%)	46 (45.1%)	
**Braf and cytoplasmic p300**			
Low Braf and low p300	22 (24.2%)	18 (17.6%)	0.001
Low Braf and high p300	16 (17.6%)	7 (6.9%)	
High Braf and low p300	33 (36.2%)	28 (27.5%)	
High Braf and high p300	20 (22.0%)	49 (48.0%)	
	**Ulceration status**		
	Absent	Present	
**Braf**			
Low	53 (36.8%)	10 (20.4%)	0.034
High	91 (63.2%)	39 (79.6%)	
**Nuclear p300**			
Low	55 (38.2%)	21 (42.9%)	0.564
High	89 (61.8%)	28 (57.1%)	
**Cytoplasmic p300**			
Low	79 (54.9%)	22 (44.9%)	0.223
High	65 (45.1%)	27 (55.1%)	
**Braf and nuclear p300**			
Low Braf and low p300	18 (12.5%)	6 (12.2%)	0.199
Low Braf and high p300	35 (24.3%)	5 (10.2%)	
High Braf and low p300	37 (25.7%)	15 (30.6%)	
High Braf and high p300	54 (37.5%)	23 (46.9%)	
**Braf and cytoplasmic p300**			
Low Braf and low p300	32 (20.8%)	8 (16.3%)	0.119
Low Braf and high p300	21 (13.6%)	2 (4.1%)	
High Braf and low p300	47 (30.5%)	14 (28.6%)	
High Braf and high p300	54 (35.1%)	25 (51.1%)	

*- χ^2^ test.

### Combination of Braf and p300 in the diagnosis of melanoma

**Figure 2:**
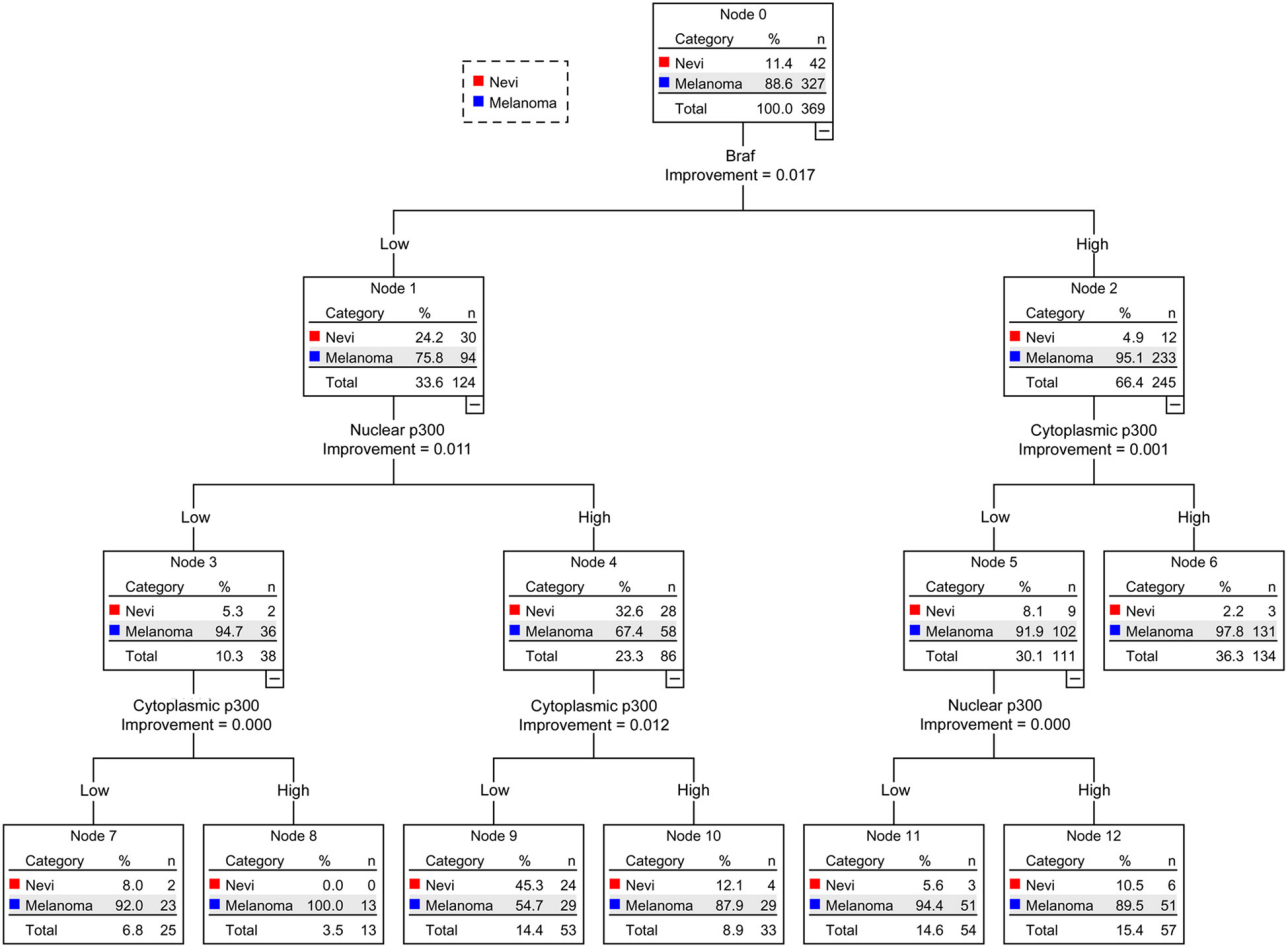
**Classification and Regression tree for differentiating nevi from melanoma using Braf, nuclear p300 and cytoplasmic p300 expression.** Nevi samples include both normal and dysplastic nevi cases. Melanoma samples include both primary and metastatic melanoma cases. ‘n’ indicates the number of samples and ‘%’ indicates the percentage of samples available at the respective node. Improvement is an indicator of separation achieved by the application of the respective marker to classify the parent node.

**Figure 3:**
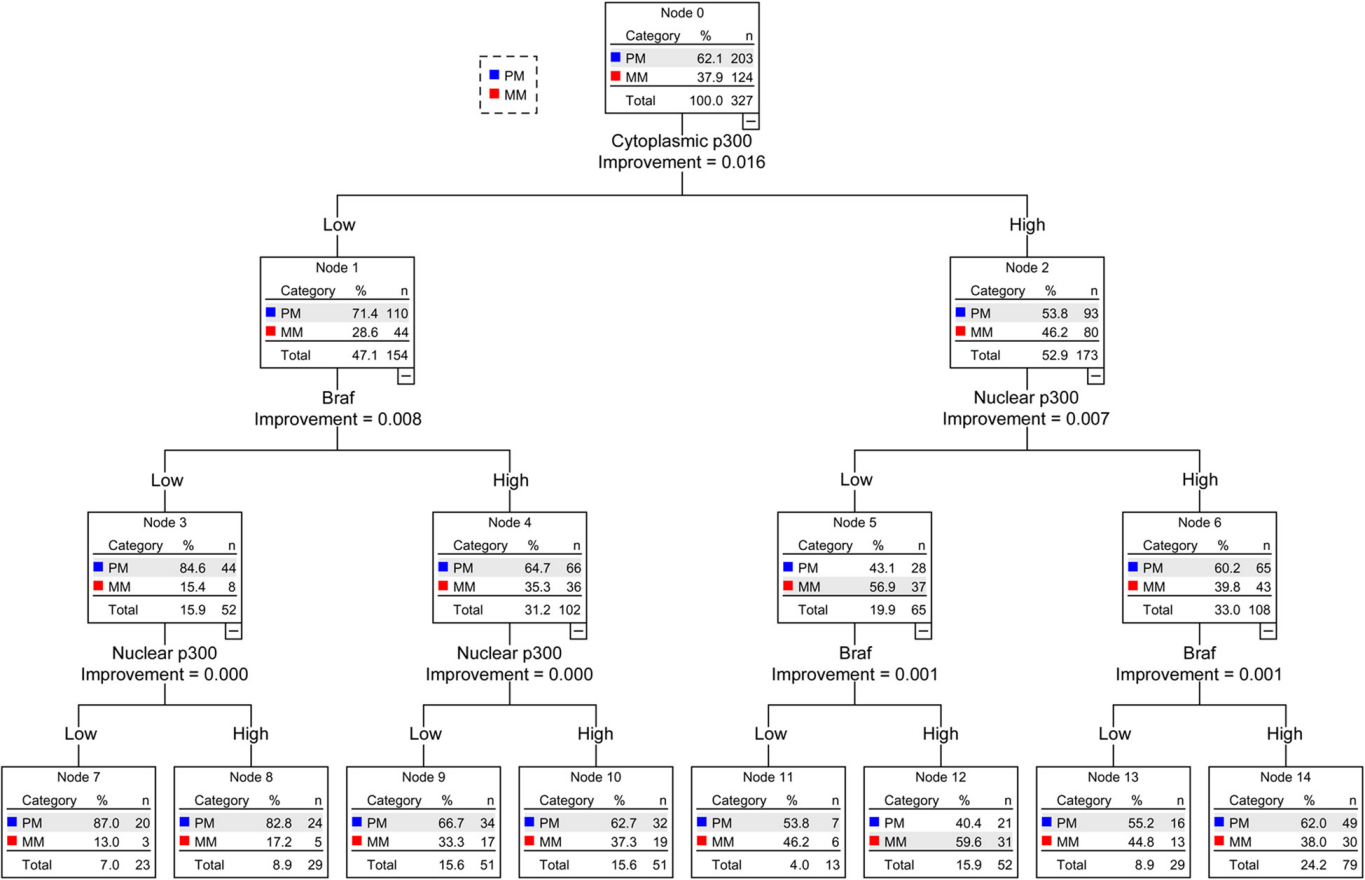
**Classification and Regression tree for differentiating primary melanoma from metastatic melanoma using Braf, nuclear p300 and cytoplasmic p300 expression.** PM, primary melanoma, includes AJCC stages I and II cases. MM, metastatic melanoma, includes stages III and IV. ‘n’ indicates the number of samples and ‘%’ indicates the percentage of samples available at the respective node. Improvement is an indicator of separation achieved by the application of the respective marker to classify the parent node.

Since we found Braf and p300 to be significantly associated with markers of advanced melanoma stages, we asked if a combination of Braf and p300 expression could be used to separate nevi from melanoma in skin biopsies. Classification and regression tree (CRT) analysis of the patient expression data was previously shown to be useful in differentiating nevi and melanoma [16]. We categorized the nevi and melanoma values as dependent variables and Braf, nuclear p300 and cytoplasmic p300 expression as independent variables, and performed CRT analysis on the data. As seen in Figure 2, Braf expression was the best marker to predict melanoma cases, followed by cytoplasmic p300 expression and nuclear p300 expression. We then used CRT analysis to test if the combination of Braf and p300 could be used to classify the primary melanoma cases and metastatic melanoma cases. As seen in Figure 3, cytoplasmic p300 expression was the best marker to separate the primary melanoma from metastatic melanoma cases, which could be further classified, using Braf and nuclear p300 expression.

### Combination of Braf and p300 in patient prognosis

**Figure 4:**
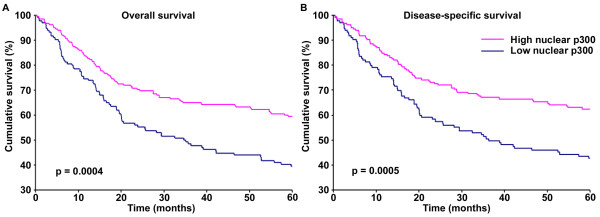
**Nuclear p300 expression and 5-year patient survival.** Kaplan-Meier survival analyses of correlation between nuclear p300 expression and 5-year overall **(A)** and disease-specific **(B)** survival of melanoma patients. The cases with low nuclear p300 expression are represented by ‘blue’ line and the cases with high expression are represented by ‘pink’ line.

**Figure 5:**
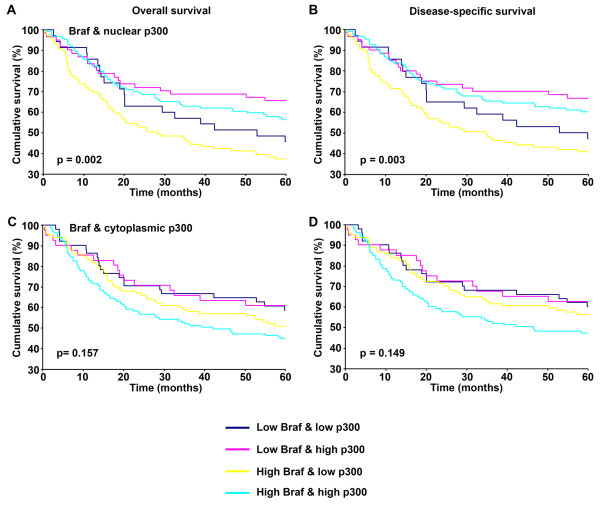
**Braf and p300 expression and 5-year patient survival.** Kaplan-Meier survival analyses of correlation between Braf & p300 expression and 5-year overall (left panels) and disease-specific (right panels) survival of melanoma patients. **(A and B)** correlation between Braf and nuclear p300 expression, and patient survival. **(C and D)** correlation between Braf and cytoplasmic p300 expression, and patient survival. Blue line represents the cases with low Braf and low p300; pink line, cases with low Braf and high p300; yellow line, high Braf and low p300; sky blue line, high Braf and high p300.

In order to test the significance of Braf and p300 in patient prognosis, we analyzed the correlation between Braf and p300 expression and patient survival using Kaplan-Meier analysis. We first confirmed the previously reported association between nuclear p300 and patient survival, and then tested a combination of Braf and nuclear p300 and studied the 5-year patient survival. As seen in Figure 4A & B, patients with low nuclear p300 expression had significantly worse 5-year survival. Intriguingly, patients with high Braf and low nuclear p300 had significantly worse 5-year survival, and patients with low Braf and high nuclear p300 had better 5-year survival, indicating the opposing effects of Braf and nuclear p300 on patient survival (Figure 5A & B). On the other hand, a combination of cytoplasmic p300 and Braf expression tended to be associated with worse prognosis and the patients with high Braf and high cytoplasmic p300 had the worst 5-year overall and disease-specific survival compared to the other categories (Figure 5C & D). However, the differences were not strong enough and failed to reach statistical significance.

### Nuclear p300 expression independently regulates patient survival

We then performed multivariate Cox regression analysis to test if Braf and/or p300 expression could independently regulate the patient survival. We used AJCC staging, nuclear p300, cytoplasmic p300, and Braf expression as variables in the model. As shown in Table 4, multivariate Cox regression analysis revealed that AJCC staging and nuclear p300 were significantly associated with patient survival, whereas the association between Braf and cytoplasmic p300, and patient survival did not reach statistical significance. Our results are in line with the previously published data showing that Braf expression was not an independent prognostic factor. It was suggested that due to the close association with the AJCC stages, tumor size and ulceration status, Braf expression could not independently predict patient survival [7].

**Table 4:** Multivariate Cox regression analysis on overall and disease-specific survival of primary melanoma patients

	**Overall survival**				**Disease-specific survival**			
**Variables**	**ß**^**†**^	**SE**	**HR (95% CI)**	***p*-value**	**ß**	**SE**	**HR (95% CI)**	***p*-value**
AJCC	1.302	0.170	3.68 (2.63-5.13)	1.98 × 10^-14^	1.457	0.182	4.29 (3.01-6.13)	1.13 × 10^-14^
Braf	0.178	0.190	1.20 (0.82-1.73)	0.348	0.110	0.196	1.12 (0.76-1.64)	0.575
Nuclear p300	-0.508	0.161	0.60 (0.44-0.83)	0.002	-0.525	0.169	0.59 (0.42-0.82)	0.002
Cytoplasmic p300	0.049	0.163	1.05 (0.76-1.45)	0.764	0.091	0.171	1.10 (0.78-1.53)	0.595

Coding of variables: AJCC was coded as 1 (stages I & II) and 2 (stages (III & IV). Braf and p300 expression was coded as 1 (low staining) and 2 (high staining).

^†^β: regression coefficient.

*Abbreviations*: *SE* standard error of β, *HR* hazard ratio, *CI* confidence interval.

## Discussion

The key to successful management of melanoma includes both early and accurate diagnosis, followed by medical intervention in the form of surgery and chemotherapy. Accuracy of the diagnosis is particularly important as misdiagnosis of the melanoma patients might lead to inadequate treatment and allow spread of the disease. Melanoma is distinguished from dysplastic nevi with a fair degree of success using routine pathological examination, but ambiguous lesions could still pose problems due to the wide variation in morphologic features and because of the overlap in the clinical and histologic features between dysplastic nevi and melanoma [16,18-21]. Our results suggest that a combination of Braf and p300 expression can be used for differentiating melanoma from nevi. The protocol for immunohistochemical staining of the tissue samples is a simple technique to perform and can give results relatively fast [22]. Since the expression of only two markers is needed to completely separate nevi from melanoma, the experimental costs are also relatively small. Our study could thus be used to develop a practical protocol, which would complement routine pathological examination and provide a clarification when tissue sections show overlapping morphologic and histologic features.

Despite significant progress in the identification of molecular pathways that drive tumorigenesis, melanoma still poses a challenge to the scientific community. Owing to its notorious resistance to chemotherapy, patients with malignant melanoma have limited treatment options and have a poor prognosis. Although, vemurafenib, a Braf^V600E^ specific inhibitor, showed impressive results in terms of response rate and progression free survival, the responses are mostly short-lived as seen by development of resistance in nearly every case [23-25]. Several strategies to increase the effectiveness, like combining Braf inhibitors with MEK1/2 inhibitors or small molecule inhibitors of the PI-3 kinase pathway, are in various stages of clinical studies, but it is too early to predict their clinical efficacy [6,25].

Our results from patient survival show that patients with low Braf and high nuclear p300 expression have better survival, hinting at the benefits of simultaneously targeting Braf and nuclear p300 in treatment of melanoma. Data from our previous study showed that though cytoplasmic p300 expression was significantly associated with clinico-pathologic characteristics of melanoma, only nuclear p300 had prognostic significance [10]. Even in the present study, cytoplasmic p300 expression was only informative during the diagnosis part of the analysis but was not a significant prognostic factor (Table 4). Besides, the major site of activity of p300 is in the nucleus where it regulates critically important processes like transcription and DNA repair [26-28]. Interestingly, loss of another well known histone acetyltransferase, TIP60, was reported to be associated with worse prognosis in melanoma patients [29]. We therefore think that combining Braf inhibitors with HDAC inhibitors might be beneficial in the chemotherapy of melanoma. Strikingly, two HDAC inhibitors, vorinostat (Merck) and romidepsin (Gloucester Pharmaceuticals), which reportedly showed inhibitory effects on melanoma growth, were approved by the US FDA for the treatment of cutaneous T-cell lymphoma [30-34]. A combination of tyrosine kinase & C-Raf inhibitor, Sorafenib and vorinostat is currently being studied in the treatment of advanced cancers [35], but we could not find any studies performed using a combination of B-raf inhibitors and vorinostat or romidepsin. Our findings encourage further research on the potential improved efficacy of coadministration of Braf and HDAC inhibitors.

Another finding of our study is the inverse correlation between Braf and nuclear p300 and direct correlation between Braf and cytoplasmic p300 expression which suggests possible cross-talk between Braf and p300. Previous studies showed that phosphorylation of p300 could differentially regulate its activity and protein stability [36,37]. For example, while protein kinase C (PKC) and salt inducible kinase 2 mediated phosphorylation at serine-89 was reported to inhibit the HAT activity [38,39], Akt mediated phosphorylation at serine-1834, serine-2279, serine-2315, and serine-2366 was shown to enhance the HAT activity of p300 [40-42]. Along those lines, Akt and ERK2 mediated phosphorylation was shown to stabilize p300 protein levels, but phosphorylation by mitogen activated protein kinase (MAPK) resulted in degradation of the p300 protein [11,12,36,40,43]. However, none of the studies have so far focused on the effect of phosphorylation on intracellular distribution of p300. Our findings point to the possible phosphorylation and altered localization of p300 by Braf/MAPK signaling, which needs further investigation.

While our database was relatively large with details of several clinical characteristics, further studies are warranted before drawing firm conclusions on the benefits of combined Braf and HDAC inhibitors. Though the significance of finding a correlation in patient biopsies cannot be underestimated, evidence from studies at the cellular level is needed to convincingly establish the relationship between Braf and p300. Furthermore, we did not have enough cases with information on the status of Braf mutations, so we were unable to analyze the potential correlation between Braf^V600E^ and p300.

## Conclusions

Our study elucidates the cross talk between Braf and p300 in melanoma and suggests that Braf might negatively regulate the accumulation of p300 in the nucleus and promote the cytoplasmic localization of p300. We also show that using a combination of Braf and p300 expression, it is possible to separate nevi and melanoma samples, and primary and metastatic melanoma samples. We show that patients with low Braf and high p300 expression have better prognosis, suggesting the possibility of combining Braf and HDAC inhibitors in melanoma treatment.

## Competing interests

The authors declare that they have no competing interests.

## Authors’ contributions

Conceived and designed the project: AR, analyzed the data: MB, MM, GA, GL, GZ, AR, and KM, wrote the manuscript: AR, KM and MB. All authors read and approved the final manuscript.

## Pre-publication history

The pre-publication history for this paper can be accessed here:

http://www.biomedcentral.com/1471-2407/14/398/prepub

## References

[B1] Miller AJ Mihm MC Jr Melanoma N Engl J Med 2006 355 1 51 65 10.1056/NEJMra052166 16822996

[B2] Rastrelli M Alaibac M Stramare R Chiarion Sileni V Montesco MC Vecchiato A Campana LG Rossi CR Melanoma m (zero): diagnosis and therapy ISRN Dermatol 2013 2013 616170 23691346 10.1155/2013/616170 PMC3649440

[B3] Siegel R Naishadham D Jemal A Cancer statistics, 2013 CA Cancer J Clin 2013 63 1 11 30 10.3322/caac.21166 23335087

[B4] Turner RM Bell KJ Morton RL Hayen A Francken AB Howard K Armstrong B Thompson JF Irwig L Optimizing the frequency of follow-up visits for patients treated for localized primary cutaneous melanoma J Clin Oncol 2011 29 35 4641 4646 10.1200/JCO.2010.34.2956 22067399

[B5] Bilimoria KY Raval MV Bentrem DJ Wayne JD Balch CM Ko CY National assessment of melanoma care using formally developed quality indicators J Clin Oncol 2009 27 32 5445 5451 10.1200/JCO.2008.20.9965 19826131

[B6] Finn L Markovic SN Joseph RW Therapy for metastatic melanoma: the past, present, and future BMC Med 2012 10 23 10.1186/1741-7015-10-23 22385436 PMC3308914

[B7] Safaee Ardekani G Jafarnejad SM Khosravi S Martinka M Ho V Li G Disease progression and patient survival are significantly influenced by BRAF protein expression in primary melanoma Br J Dermatol 2013 169 2 320 328 10.1111/bjd.12351 23550516

[B8] Safaee Ardekani G Jafarnejad SM Tan L Saeedi A Li G The prognostic value of BRAF mutation in colorectal cancer and melanoma: a systematic review and meta-analysis PLoS One 2012 7 10 e47054 10.1371/journal.pone.0047054 23056577 PMC3467229

[B9] Roth SY Denu JM Allis CD Histone acetyltransferases Annu Rev Biochem 2001 70 81 120 10.1146/annurev.biochem.70.1.81 11395403

[B10] Rotte A Bhandaru M Cheng Y Sjoestroem C Martinka M Li G Decreased expression of nuclear p300 is associated with disease progression and worse prognosis of melanoma patients PLoS One 2013 8 9 e75405 10.1371/journal.pone.0075405 24098694 PMC3787094

[B11] Poizat C Puri PL Bai Y Kedes L Phosphorylation-dependent degradation of p300 by doxorubicin-activated p38 mitogen-activated protein kinase in cardiac cells Mol Cell Biol 2005 25 7 2673 2687 10.1128/MCB.25.7.2673-2687.2005 15767673 PMC1061628

[B12] Wang SA Hung CY Chuang JY Chang WC Hsu TI Hung JJ Phosphorylation of p300 increases its protein degradation to enhance the lung cancer progression Biochim Biophys Acta 2014 1843 6 1135 1149 10.1016/j.bbamcr.2014.02.001 24530506

[B13] Zhang Z Chen G Cheng Y Martinka M Li G Prognostic significance of RUNX3 expression in human melanoma Cancer 2011 117 12 2719 2727 10.1002/cncr.25838 21656750

[B14] Chen G Cheng Y Zhang Z Martinka M Li G Cytoplasmic Skp2 expression is increased in human melanoma and correlated with patient survival PLoS One 2011 6 2 e17578 10.1371/journal.pone.0017578 21386910 PMC3046256

[B15] Remmele W Stegner HE [Recommendation for uniform definition of an immunoreactive score (IRS) for immunohistochemical estrogen receptor detection (ER-ICA) in breast cancer tissue] Pathologe 1987 8 3 138 140 3303008

[B16] Zhang G Li G Novel multiple markers to distinguish melanoma from dysplastic nevi PLoS One 2012 7 9 e45037 10.1371/journal.pone.0045037 23028750 PMC3459895

[B17] Balch CM Gershenwald JE Soong SJ Thompson JF Atkins MB Byrd DR Buzaid AC Cochran AJ Coit DG Eggermont AM DS Flaherty KT Gimotty PA Kirkwood JM McMasters KM Mihm MC Jr Morton DL Ross MI Sober AJ Sondak VK Final version of 2009 AJCC melanoma staging and classification J Clin Oncol 2009 27 36 6199 6206 10.1200/JCO.2009.23.4799 19917835 PMC2793035

[B18] Pellacani G Longo C Ferrara G Cesinaro AM Bassoli S Guitera P Menzies SW Seidenari S Spitz nevi: In vivo confocal microscopic features, dermatoscopic aspects, histopathologic correlates, and diagnostic significance J Am Acad Dermatol 2009 60 2 236 247 10.1016/j.jaad.2008.07.061 19091443

[B19] Duffy K Grossman D The dysplastic nevus: from historical perspective to management in the modern era: part II. Molecular aspects and clinical management J Am Acad Dermatol 2012 67 1 19 e11 12 quiz 31–12 22703916 10.1016/j.jaad.2012.03.013 PMC3621132

[B20] Duffy K Grossman D The dysplastic nevus: from historical perspective to management in the modern era: part I. Historical, histologic, and clinical aspects J Am Acad Dermatol 2012 67 1 1 e1 16 quiz 17–18 10.1016/j.jaad.2011.02.032 22703915 PMC3625372

[B21] Rabkin MS The limited specificity of histological examination in the diagnosis of dysplastic nevi J Cutan Pathol 2008 35 Suppl 2 20 23 18976415 10.1111/j.1600-0560.2008.01131.x

[B22] Camp RL Neumeister V Rimm DL A decade of tissue microarrays: progress in the discovery and validation of cancer biomarkers J Clin Oncol 2008 26 34 5630 5637 10.1200/JCO.2008.17.3567 18936473

[B23] Chapman PB Hauschild A Robert C Haanen JB Ascierto P Larkin J Dummer R Garbe C Testori A Maio M Hogg D Lorigan P Lebbe C Jouary T Schadendorf D Ribas A O'Day SJ Sosman JA Kirkwood JM Eggermont AMM Dreno B Nolop K Li J Nelson B Hou J Lee RJ Flaherty KT McArthur GA for the BRIM-3 Study Group Improved survival with vemurafenib in melanoma with BRAF V600E mutation N Engl J Med 2011 364 26 2507 2516 10.1056/NEJMoa1103782 21639808 PMC3549296

[B24] Flaherty KT Puzanov I Kim KB Ribas A McArthur GA Sosman JA O’Dwyer PJ Lee RJ Grippo JF Nolop K Chapman PB Inhibition of mutated, activated BRAF in metastatic melanoma N Engl J Med 2010 363 9 809 819 10.1056/NEJMoa1002011 20818844 PMC3724529

[B25] Kudchadkar R Paraiso KH Smalley KS Targeting mutant BRAF in melanoma: current status and future development of combination therapy strategies Cancer J 2012 18 2 124 131 10.1097/PPO.0b013e31824b436e 22453012 PMC3314865

[B26] Iyer NG Ozdag H Caldas C p300/CBP and cancer Oncogene 2004 23 24 4225 4231 10.1038/sj.onc.1207118 15156177

[B27] Reed SH Nucleotide excision repair in chromatin: damage removal at the drop of a HAT DNA Repair (Amst) 2011 10 7 734 742 10.1016/j.dnarep.2011.04.029 21600858

[B28] Tillhon M Cazzalini O Nardo T Necchi D Sommatis S Stivala LA Scovassi AI Prosperi E p300/CBP acetyl transferases interact with and acetylate the nucleotide excision repair factor XPG DNA Repair (Amst) 2012 11 10 844 852 10.1016/j.dnarep.2012.08.001 22954786

[B29] Chen G Cheng Y Tang Y Martinka M Li G Role of Tip60 in human melanoma cell migration, metastasis, and patient survival J Invest Dermatol 2012 132 11 2632 2641 10.1038/jid.2012.193 22673729

[B30] Giannini G Cabri W Fattorusso C Rodriquez M Histone deacetylase inhibitors in the treatment of cancer: overview and perspectives Future Med Chem 2012 4 11 1439 1460 10.4155/fmc.12.80 22857533

[B31] Haigentz M Jr Kim M Sarta C Lin J Keresztes RS Culliney B Gaba AG Smith RV Shapiro GI Chirieac LR Mariadason JM Belbin TJ Greally JM Wright JJ Haddadf RI Phase II trial of the histone deacetylase inhibitor romidepsin in patients with recurrent/metastatic head and neck cancer Oral Oncol 2012 48 12 1281 1288 10.1016/j.oraloncology.2012.05.024 22748449 PMC3465519

[B32] Murakami T Sato A Chun NA Hara M Naito Y Kobayashi Y Kano Y Ohtsuki M Furukawa Y Kobayashi E Transcriptional modulation using HDACi depsipeptide promotes immune cell-mediated tumor destruction of murine B16 melanoma J Invest Dermatol 2008 128 6 1506 1516 10.1038/sj.jid.5701216 18185535

[B33] Landreville S Agapova OA Matatall KA Kneass ZT Onken MD Lee RS Bowcock AM Harbour JW Histone deacetylase inhibitors induce growth arrest and differentiation in uveal melanoma Clin Cancer Res 2012 18 2 408 416 10.1158/1078-0432.CCR-11-0946 22038994 PMC3261307

[B34] Dekker FJ Haisma HJ Histone acetyl transferases as emerging drug targets Drug Discov Today 2009 14 19–20 942 948 19577000 10.1016/j.drudis.2009.06.008

[B35] Dasari A Gore L Messersmith WA Diab S Jimeno A Weekes CD Lewis KD Drabkin HA Flaig TW Camidge DR A phase I study of sorafenib and vorinostat in patients with advanced solid tumors with expanded cohorts in renal cell carcinoma and non-small cell lung cancer Invest New Drugs 2013 31 1 115 125 10.1007/s10637-012-9812-z 22415798

[B36] Wang QE Han C Zhao R Wani G Zhu Q Gong L Battu A Racoma I Sharma N Wani AA p38 MAPK- and Akt-mediated p300 phosphorylation regulates its degradation to facilitate nucleotide excision repair Nucleic Acids Res 2013 41 3 1722 1733 10.1093/nar/gks1312 23275565 PMC3561975

[B37] Chen J Li Q Life and death of transcriptional co-activator p300 Epigenetics 2011 6 8 957 961 10.4161/epi.6.8.16065 21730760

[B38] Bricambert J Miranda J Benhamed F Girard J Postic C Dentin R Salt-inducible kinase 2 links transcriptional coactivator p300 phosphorylation to the prevention of ChREBP-dependent hepatic steatosis in mice J Clin Invest 2010 120 12 4316 4331 10.1172/JCI41624 21084751 PMC2993582

[B39] Yuan LW Gambee JE Phosphorylation of p300 at serine 89 by protein kinase C J Biol Chem 2000 275 52 40946 40951 10.1074/jbc.M007832200 11020388

[B40] Chen J Halappanavar SS St-Germain JR Tsang BK Li Q Role of Akt/protein kinase B in the activity of transcriptional coactivator p300 Cell Mol Life Sci 2004 61 13 1675 1683 15224190 10.1007/s00018-004-4103-9 PMC11138905

[B41] Huang WC Chen CC Akt phosphorylation of p300 at Ser-1834 is essential for its histone acetyltransferase and transcriptional activity Mol Cell Biol 2005 25 15 6592 6602 10.1128/MCB.25.15.6592-6602.2005 16024795 PMC1190347

[B42] Liu Y Denlinger CE Rundall BK Smith PW Jones DR Suberoylanilide hydroxamic acid induces Akt-mediated phosphorylation of p300, which promotes acetylation and transcriptional activation of RelA/p65 J Biol Chem 2006 281 42 31359 31368 10.1074/jbc.M604478200 16926151

[B43] Chen YJ Wang YN Chang WC ERK2-mediated C-terminal serine phosphorylation of p300 is vital to the regulation of epidermal growth factor-induced keratin 16 gene expression J Biol Chem 2007 282 37 27215 27228 10.1074/jbc.M700264200 17623675

